# Serotype G12 Rotaviruses, Lilongwe, Malawi

**DOI:** 10.3201/eid1501.080427

**Published:** 2009-01

**Authors:** Nigel A. Cunliffe, Bagrey M. Ngwira, Winifred Dove, Osamu Nakagomi, Toyoko Nakagomi, Arantza Perez, C. Anthony Hart, Peter N. Kazembe, Charles C.V. Mwansambo

**Affiliations:** University of Liverpool, Liverpool, UK (N.A. Cunliffe, W. Dove, O. Nakagomi, T. Nakagomi, C.A. Hart); College of Medicine, Blantyre, Malawi (B.M. Ngwira, A. Perez); Baylor College of Medicine Children's Foundation, Lilongwe, Malawi (P.N. Kazembe); Kamuzu Central Hospital, Lilongwe (C.C.V. Mwansambo); Nagasaki University, Nagasaki, Japan (O. Nakagomi, T. Nakagomi); 1 Deceased.

**Keywords:** Gastroenteritis, rotavirus, electropherotype, VP7, VP4, Malawi, dispatch

## Abstract

To assess diversity of rotavirus strains in Lilongwe, Malawi, we conducted a cross-sectional study of children with acute gastroenteritis, July 2005–June 2007. Serotype G12 was identified in 30 (5%) of 546 rotavirus-positive fecal specimens. The G12 strain possessed multiple electropherotypes and P-types, but their viral protein 7 sequences were closely related, indicating that reassortment has occurred.

Rotavirus is the leading cause of severe, acute gastroenteritis, a disease that causes dehydration and death in infants and young children worldwide (*1*); an estimated 527,000 childhood deaths occur annually (who.int/immunization_monitoring/burden/rotavirus_estimates/en/index.htm). Because of the high death rates in children, vaccination to prevent severe disease outcomes after rotavirus infection is an essential public health strategy (*2*,*3*). Currently, 2 live attenuated oral rotavirus vaccines are becoming part of childhood immunization schedules in North America, Latin America, and Europe; Phase III clinical trials are underway in Africa and Asia (*3*).

Rotaviruses are segmented, double-stranded (ds) RNA viruses that possess a triple-layered protein capsid. The 11 dsRNA segments, upon separation by electrophoresis, exhibit profiles that can be broadly categorized into long and short RNA patterns termed electropherotypes. The rotavirus outer capsid comprises 2 neutralization antigens, VP7 and VP4, which respectively define the G (for glycoprotein) and P (for protease-sensitive) serotypes. The 5 globally most common rotavirus strain types comprise long electropherotype P[8] strains possessing G1, G3, G4, or G9 specificity and short electropherotype G2P[4] strains (*4*). Rotaviruses exhibit considerable diversity, including unusual combinations of electropherotypes and serotypes (which suggests viral reassortment); globally, rare G and P types predominate in some regions (*5*). For example, we have previously described serotype G8 to be a locally prevalent serotype in Blantyre, Malawi (*6*). More recently, rotavirus serotype G12 has emerged in multiple countries (*7*). Our study assesses diversity of rotavirus strains in Lilongwe, Malawi, in anticipation of introduction of a rotavirus vaccine in this country.

## The Study

This 2-year, cross-sectional study was undertaken at Kamuzu Central Hospital in Lilongwe from July 2005 through June 2007. Children <5 years of age with acute gastroenteritis who received oral and/or intravenous rehydration therapy, were enrolled after parents or guardians gave written, informed consent. Study participants included outpatients and inpatients. A fecal sample was collected from each case-patient and stored at –80°C until rotavirus detection and characterization were undertaken.

Group A rotavirus antigen was detected by a commercial ELISA performed according to the manufacturer’s instructions (Rotaclone; Meridian Diagnostics, Cincinnati, OH, USA). Among rotavirus antigen–positive specimens, specimens that exhibited color intensity at least equal to the positive control provided with the Rotaclone kit were selected for further strain characterization. Genotyping by multiplex, heminested reverse transcription-PCR (RT-PCR) was undertaken as previously described (*6*). Specimens that remained G nontypeable were analyzed by using a G12-typing primer (*8*), and those that could not be P-typed were further examined by a degenerate P[8] primer (*9*). Serotype G12 strains were further examined by polyacrylamide gel electrophoresis (PAGE) of rotavirus dsRNA followed by silver staining to determine the rotavirus electropherotype as previously described (*10*).

Full-length VP7 genes of G12 strains representing distinct electropherotypes were obtained by RT-PCR by using primers Beg 9 and End 9 (*11*). Amplification products were purified by using Minispin columns (Amersham, Buckinghamshire, UK) and sequenced by Cogenics Inc. (Hope End, Essex, UK). GenBank accession numbers representing the gene sequence encoding VP7 of each Malawi G12 strain examined are as follows (for each rotavirus strain, the prefix KCH is used to denote Kamuzu Central Hospital): KCH344 (EU573776); KCH1050 (EU573777); KCH1051 (EU573778); KCH1124 (EU573779); KCH569 (EU573780); KCH1074 (EU573781); KCH602 (EU573782).

Rotavirus was detected in 578 (38%) of 1,522 specimens, of which 419 (39%) of 1,070 were from inpatients and 159 (35%) of 452 were from outpatients. A total of 546 rotavirus- positive specimens were further characterized. The most commonly detected strain types included G1P[8] (47%), G8P[8] (12%), G1P[6] (10%), G8P[6] (7%), G8P[4] (6%), and G12 P[6] (4%) (Table). A total of 48 specimens (9%) could not be assigned a G and/or P type. Overall, G1 was the most common G-type (58%), followed by G8 (29%) and G12 (5%); P[8] was the most common P-type (64%) followed by P[6] (23%) and P[4] (7%).

**Table:** Distribution of rotavirus G and P types in Lilongwe, Malawi, July 2005–June 2007*

Type	G1	G2	G4	G8	G9	G12	G1 + G8	G8 + G9	GNT	Total
P[4]	1	5		32		1				39
P[6]	55	1	1	39	3	22	1		3	125
P[8]	255			64	9	5		2	12	347
P[6] + P[8]	1			1						2
P[NT]	6			20	1	2			4	33
Total	318	6	1	156	13	30	1	2	19	546

*NT, nontypeable.

**Figure 1:**
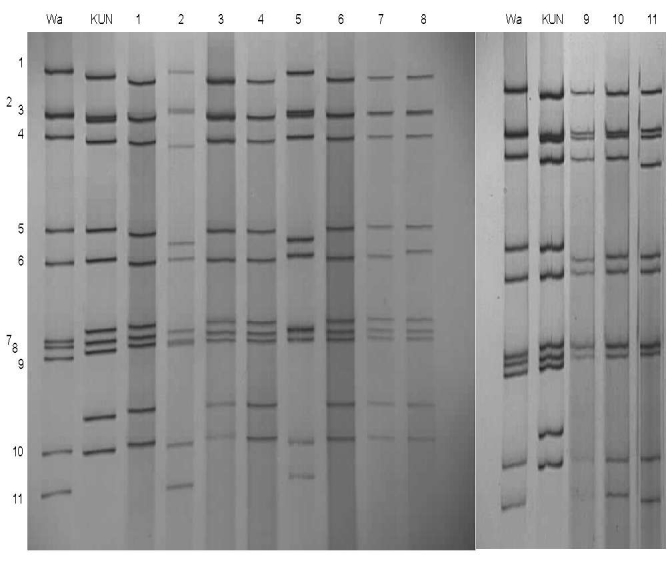
Polyacrylamide gel electrophoresis and silver staining of rotavirus double-stranded RNA of representative serotype G12 strains from Lilongwe, Malawi. RNA segments are indicated to the left. Strains Wa (long electropherotype) and KUN (short electropherotype) are controls. Field strains, designated electropherotype profiles, and P types are as follows: Lane 1, KCH958 short – profile S1, P[6]; lane 2, KCH1120, long – profile L1, P[6]; lane 3, KCH1124, short – profile S1, P[6]; lane 4, KCH1050, short – profile S1, P[6]; lane 5, LOP286, long – profile L2, P[6]; lane 6, LOP523, short – profile S1, P[6]; lane 7, KCH944, short – profile S1, P[6]; lane 8, KCH1074, short – profile S2, P[6]; lane 9, KCH569, long – profile L2, P[6]; lane 10, KCH602, long – profile L2, P[6]; lane 11, KCH344, long – profile L3, P[8].

**Figure 2:**
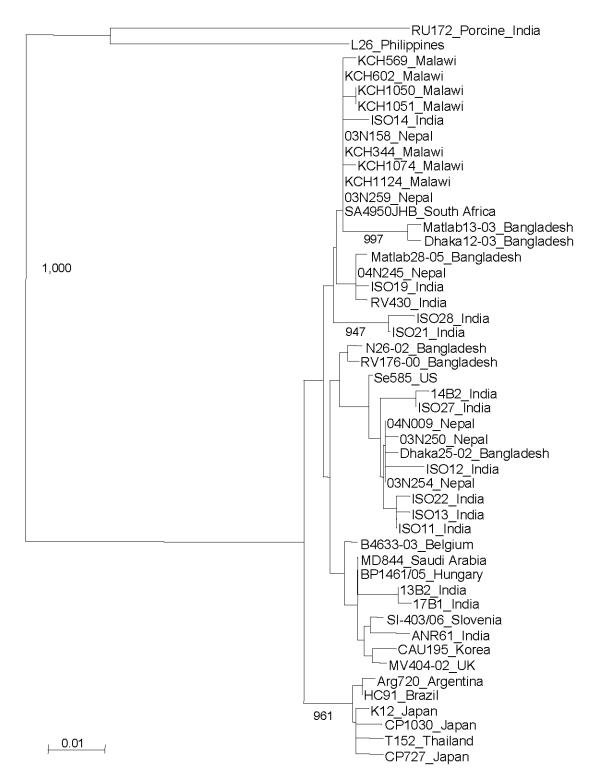
Phylogenetic tree based on VP7 nucleotide sequences from representative Malawi G12 strains and G12 strains deposited in DNA databases. Strain designations are followed by country of origin. Malawi strain KCH1051 is genotype G12P[8] and has an indeterminate RNA profile. Horizontal lengths are proportional to the genetic distance calculated with the Kimura 2-parameter method. Number adjacent to the node represents the bootstrap value of 1,000 replicates and values <80% are not indicated. Scale bar shows genetic distance expressed as nucleotide substitutions per site.

All but 2 G12 strains were detected in the second year of the study (July 2006 through June 2007). The G12 strains were associated with VP4 types P[6] (n = 22), P[8] (n = 5), P[4] (n = 1), and P[NT] (n = 2) and were investigated further by electropherotyping and nucleotide sequencing. Among the 30 G12 strains examined, 23 (77%) produced an identifiable electropherotype. Among G12P[6] strains, 11 displayed short electropherotypes (2 distinct patterns were recognized, with 1 predominant), and 9 strains had long electropherotypes (with 2 distinct patterns recognized). A single P[8] strain, KCH344, had a recognizable but distinct electropherotype (Figure 1). Two G12P[NT] strains possessed, respectively, short and long RNA profiles (data not shown). The single G12P[4] strain had no visible RNA after PAGE. Full-length VP7 sequences that could be successfully obtained by RT-PCR from G12 strains representing distinct electropherotypes were compared with each other and with published G12 sequences from elsewhere in the world (Figure 2). The VP7 genes of 7 Malawian G12 strains shared >99% nucleotide identity with each other, despite possessing a variety of electropherotypes and P-types, and were most closely related to recently identified G12 strains detected in Nepal, India, and South Africa.

## Conclusions

Rotavirus was identified as a leading cause of gastroenteritis among infants and young children seeking hospital care in Lilongwe, Malawi; the virus was detected in 38% of all case-patients. The G1P[8] strain, globally the most common rotavirus strain type, was also the most commonly identified rotavirus strain in Lilongwe, comprising 47% of all characterized strains. Serotype G8, first identified in Blantyre, Malawi, in the late 1990s in association with the P[6] and P[4] VP4 types and considered to have arisen by viral reassortment from a bovine origin (*6*,*12*), was detected in 29% of strains in association with P-types P[8], P[6], and P[4]. The G8P[8] strain, comprising 41% of all G8 strains in this collection, is gaining increasing global recognition as an emerging strain type (*13*). In this study, we have also identified the globally common serotype G2, in association with VP4 types P[4] and P[6]. Notably, rotaviruses bearing the P[6] VP4 type comprised 23% of all characterized strains, thus providing further evidence of its prominence in Africa (*4*) (Table).

We have identified in Malawi the globally emerging rotavirus serotype G12, which was detected in 5% of all specimens. The single previous description of serotype G12 from the African continent was from Johannesburg, South Africa (*14*). G12 rotaviruses, first identified in the Philippines in 1987, have emerged over the past few years in numerous countries worldwide (*7*).

In our study, G12 was associated predominantly with the P[6] VP4 type and less commonly with P[8] and P[4]. Multiple electropherotypes were demonstrated within P[6] strains, including long and short profiles. In contrast, the VP7 sequence of strains representing each electropherotype and P-type were closely related, sharing >99% nucleotide identity. These findings suggest the introduction of a single G12 VP7 gene into local rotavirus strains, which have subsequently undergone reassortment to generate G12 strains harboring a constellation of electropherotypes and P-types. Similar diversity among G12 strains has recently been described in Nepal (*8*,*15*). The critical role that viral reassortment has played in generating extensive genetic diversity among G12 rotaviruses in Lilongwe mirrors the evolutionary mechanisms that have led to the global emergence of this serotype (*7*). The close relationship of the VP7 genes of Lilongwe G12 strains with G12 strains from Nepal and India suggests an origin in Asia, a hypothesis proposed by Rahman et al. (*7*).

Two current rotavirus vaccines, Rotarix (GSK Biologicals, Rixensart, Belgium) and RotaTeq (Merck & Co., Whitehouse Station, NJ, USA) offer the potential to greatly reduce childhood deaths from rotavirus gastroenteritis (*2*,*3*). The monovalent vaccine Rotarix comprises a human G1P[8] rotavirus strain, whereas the pentavalent vaccine RotaTeq is a human-bovine reassortant vaccine comprising human serotypes G1, G2, G3, G4, and P[8] on a bovine strain background. Although both vaccines are highly effective in preventing severe rotavirus gastroenteritis in North America, Latin America, and Europe, their efficacy in countries harboring a wider diversity of strain types is yet to be fully established (*2*,*3*). Other rotavirus proteins (e.g., VP6 and NSP4) may play a role in the protective immunity against rotavirus infection; G12P[6] strains detected in the current study share neither G- nor P-type with either of the 2 current vaccines and could theoretically challenge vaccine efficacy. Continued surveillance for serotype G12 in Malawi and elsewhere in Africa is needed, given the propensity of this emerging serotype to rapidly spread and establish itself within populations (*7*,*8*).
